# Subclinical hypothyroidism, focusing on carpal tunnel syndrome and peroneal neuropathy at the fibular head: a prospective case-control study

**DOI:** 10.1590/1516-3180.2024.0280.R1.07032025

**Published:** 2025-06-02

**Authors:** Yunus Coşkun, Halit Fidanci

**Affiliations:** I Department of Internal Medicine, Adana City Training and Research Hospital, University of Health Sciences, Adana, Türkiye.; II Department of Neurology, Division of Clinical Neurophysiology, Adana City Training and Research Hospital, University of Health Sciences, Adana, Türkiye.

**Keywords:** Carpal tunnel syndrome, Electrodiagnosis, Hypothyroidism, Nerve conduction studies, Peroneal neuropathy, Median nerve injury at wrist, Peroneal nerve injury at the fibular head, Subclinical hypothyroidism

## Abstract

**BACKGROUND:**

Peripheral nerves may be affected in subclinical hypothyroidism (SH).

**OBJECTIVES:**

This study aimed to investigate the presence of common entrapment mononeuropathies in patients with SH.

**DESIGN AND SETTING:**

A prospective case-control study conducted between September 2022 and November 2023 at Adana City Training and Research Hospital, Adana, Türkiye.

**METHODS:**

SH patients without neurological complaints and healthy individuals over the age of 18 were included. Serum levels of free T3, free T4, thyroid-stimulating hormone (TSH), thyroid peroxidase (TPO) antibody, and creatine kinase (CK) were measured. All participants underwent nerve conduction studies of the upper and lower extremities.

**RESULTS:**

Thirty patients with SH and 40 healthy individuals were included in the study. The percentage reduction in compound muscle action potential (CMAP) amplitude across the knee segment was 2.8 ± 3.5% in healthy individuals and 6.7 ± 6.6% in SH patients (P = 0.017). Apart from this significant difference, other nerve conduction study findings did not differ between the two groups. A positive correlation was observed between CK levels and the percentage reduction in peroneal nerve CMAP amplitude across the knee segment (P = 0.021, r = 0.421). Additionally, there was a positive correlation between TPO antibody levels and F-wave latency in both the median and ulnar nerves (P = 0.028 r = 0.400/P = 0.005 r = 0.501). Electrodiagnostic evaluations revealed carpal tunnel syndrome (CTS) in four patients, peroneal neuropathy at the fibular head (PNFH) in four patients, and ulnar neuropathy at the elbow (UNE) in one patient.

**CONCLUSION:**

This study suggests that patients with SH may develop subclinical CTS and PNFH, but not UNE. Accordingly, it highlights the importance of avoiding risk factors that may contribute to the development of CTS and PNFH. Serum CK and TPO antibody levels may be useful in monitoring subclinical neuropathy in SH.

## INTRODUCTION

Peripheral nerves can become compressed while passing through narrow structures, leading to entrapment mononeuropathies, which manifest clinically as symptoms ranging from paresthesias to weakness.^1,2,3^ Carpal tunnel syndrome (CTS) and ulnar neuropathy at the elbow (UNE) are common forms of upper extremity entrapment mononeuropathies. Meanwhile, peroneal neuropathy at the fibular head (PNFH) is a prevalent lower extremity entrapment neuropathy.^2^ Many factors, including certain occupations and chronic diseases like diabetes mellitus and thyroid disease, are associated with these neuropathies.^1–4^ Neuropathy linked to thyroid conditions can lead to reduced deep tendon reflexes, muscle weakness, neuropathic pain, and paresthesias, significantly affecting daily activities. Although the precise pathophysiology of neuropathy in thyroid diseases is not fully understood, reductions in adenosine triphosphate activity and metabolic alterations are believed to play a role. These changes may cause damage to the nerve cell body, myelin sheath, or axons.^6,7^ While it is well-established that thyroid diseases are risk factors for neuropathies such as CTS and polyneuropathy, their relationship with UNE or PNFH remains less understood.^4,5,6,7^

## OBJECTIVE

Electrophysiological tests are crucial in diagnosing entrapment mononeuropathies, providing essential information for both diagnosis and prognosis.^8,9^ In motor and sensory nerve conduction studies, common electrophysiological abnormalities linked to thyroid diseases include reduced potential amplitudes and moderately slowed conduction velocities.^7^ Notably, these abnormalities have been observed in patients with subclinical hypothyroidism (SH) who do not have neuropathic symptoms, suggesting that thyroid diseases might exert a subclinical impact on peripheral nerves.^10^ Research has been conducted to determine whether there is an increased predisposition to CTS, UNE and PNFH in patients with SH who do not present neuropathic complaints.

## METHODS

### Subjects and study design

Patients with SH displaying normal thyroid function and healthy individuals who presented to the Internal Medicine Department of the University of Health Sciences Adana City Training and Research Hospital (ACTRH) between September 2022 and November 2023 were included in this study. Ethics committee approval for this prospective case-control study was obtained from the ACTRH Clinical Research Ethics Committee (approval number 1934/105/2022). Written consent was secured from all participants. Neurological examinations were conducted on both SH patients and healthy individuals, and the levels of serum free T3 (T3), free T4 (T4), thyroid stimulating hormone (TSH), thyroid peroxidase (TPO) antibody, and creatine kinase (CK) were assessed. SH was considered in patients exhibiting the following characteristics:^11,12,13^ 1) positivity for thyroglobulin antibody and/or thyroid peroxidase antibody; 2) thyroid ultrasonography results consistent with SH. Patients were required to have been diagnosed with SH for at least one month. Both SH patients and healthy individuals were excluded from the study if they exhibited any of the following: 1) sensory abnormalities or weakness in the extremities; 2) abnormalities in neurological examinations; 3) any neurodegenerative disease; 4) conditions or diseases predisposing to neuropathy, such as diabetes mellitus; 5) abnormal levels of T3, T4, or TSH. Additionally, healthy individuals with abnormal levels of TPO antibody, thyroglobulin antibody, or CK were also excluded from the study. Neurophysiological tests were conducted on all participants at the University of Health Sciences ACTRH Neurophysiology Laboratory. These tests, along with the assessment of serum T3, T4, TSH, TPO antibody, and CK levels, were all performed on the same day.

### Electrodiagnostic tests

Nerve conduction studies were performed on one upper and one lower extremity of each participant using the Cadwell Sierra Summit EMG unit (Cadwell Laboratories, Kennewick, Washington, USA). Electrodiagnostic tests were conducted only if the extremity temperature was above 32°C; otherwise, the limb was warmed prior to testing. For motor and sensory nerve conduction studies, the high- and low-pass filter settings were 20 Hz–10 kHz and 20 Hz–2 kHz, respectively. Surface electrodes were used for both stimulation and recording, and stimulation was applied supramaximally. Sweep speed and sensitivity were set to 5 ms/2 mV for motor studies and 1 ms/10 μV for sensory studies. Compound nerve action potential (CNAP) and compound muscle action potential (CMAP) amplitudes were calculated by measuring from peak to peak. Median and ulnar nerve CNAPs were recorded antidromically from the 2nd and 5th fingers, respectively. Sural and superficial peroneal nerve CNAPs were also obtained antidromically. Sensory nerve conduction velocity (NCV) was calculated using peak latency for the median, ulnar, and sural nerves, and onset latency for the superficial peroneal nerve. Stimulation sites for motor nerve conduction studies were as follows: median nerve—wrist to elbow; ulnar nerve—wrist, below elbow, and above elbow; peroneal nerve—ankle, below the fibular head, and popliteal fossa; posterior tibial nerve—ankle to popliteal fossa. Peroneal nerve CMAPs were recorded from both the extensor digitorum brevis and tibialis anterior muscles. Among the ten F-wave responses recorded, the one with the shortest latency was selected for analysis.

### Identifying entrapment neuropathy

CTS was considered electrophysiologically if any of the following criteria were met:^14,15,16^ 1) median sensory NCV across the 2nd finger–wrist segment < 39 m/s; 2) In addition to slowed median sensory NCV, median nerve CMAP distal latency > 3.7 ms; 3) If the median nerve CNAP could not be obtained, and the median nerve CMAP distal latency was > 3.7 ms.

UNE was diagnosed electrophysiologically if any of the following findings were present:^16,17,18^ 1) Ulnar motor NCV across the elbow segment < 43 m/s; 2) CMAP amplitude reduction across the elbow segment > 20%; 3) A difference in ulnar motor NCV > 15 m/s between the forearm and elbow segments.

PNFH was diagnosed electrophysiologically if any of the following criteria were met:^17,19,20^ 1) peroneal motor NCV < 40.1 m/s (recorded from the extensor digitorum brevis [EDB]) or < 41 m/s (recorded from the tibialis anterior [TA]) across the knee segment; 2) CMAP amplitude decrease > 25% across the knee segment; 3) peroneal motor NCV difference > 6 m/s between the leg segment and the knee segment.

### Statistical analysis

Numerical data are presented with the mean ± standard deviation (SD). Frequency and percentage were used to define categorical variables. Pearson’s chi-square test was used to compare categorical data between two groups. Numerical data were compared between the two groups with the Mann-Whitney U test. Correlation analyses were performed using Spearman’s correlation test. It was considered statistically significant if P < 0.05. SPSS 22.0 program was used for statistical analysis.

## RESULTS

Thirty-three patients with SH were initially assessed; however, three were excluded due to the presence of diabetes mellitus and polyneuropathy, confirmed by nerve conduction studies. Two of these three patients also had electrophysiological findings consistent with CTS. Ultimately, 30 SH patients (4 male, 26 female) and 40 healthy individuals (7 male, 33 female) were included in the study (P = 0.747 for sex comparison). The mean ages of the SH patients and healthy individuals were 39.9 ± 12.6 years (range: 18–65) and 38.5 ± 11.4 years (range: 18–64), respectively (P = 0.557). The mean body mass index (BMI) was 30.3 ± 5.7 kg/m^2^ (range: 21.5–42.9) for SH patients and 25.3 ± 3.8 kg/m^2^ (range: 17.9–27.4) for healthy individuals (P < 0.001). The mean duration of SH was 7.2 ± 7.6 months (range: 1–30), with 21 patients (70%) having a disease duration of less than 6 months. All SH patients were receiving levothyroxine therapy. The mean values of laboratory parameters in SH patients were as follows: T3, 5.4 ± 7.9 ng/dL; T4, 0.9 ± 0.2 ng/dL; TSH, 3.9 ± 2.7 mIU/L; TPO antibody, 485.8 ± 544.7 IU/mL; and CK, 94.7 ± 46.3 U/L. T3, T4, and TSH levels were within the normal range in both SH patients and healthy individuals.

**Figure 1:**
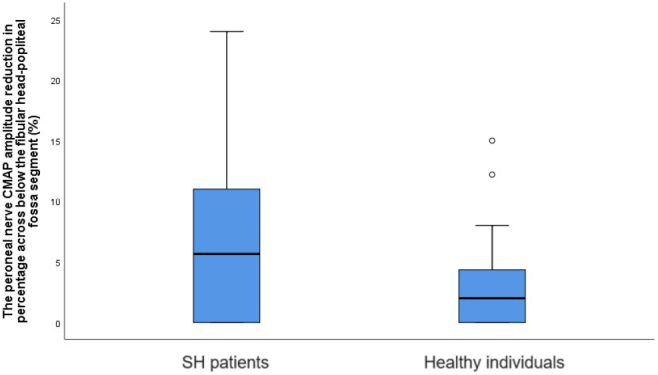
Comparison of peroneal nerve CMAP amplitude reduction in percentage across below the fibular head-popliteal fossa segment between healthy individuals and SH patients.

**Figure 2:**
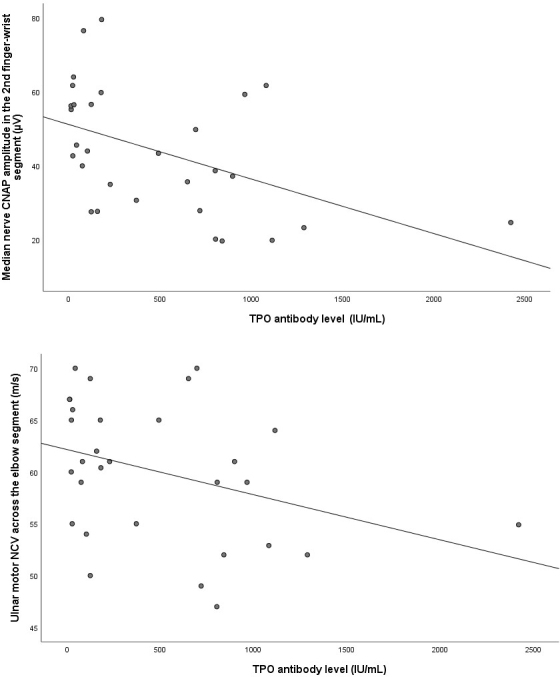
Correlation between TPO antibody level and median nerve CNAP amplitude in the 2nd finger-wrist segment/ulnar motor NCV across the elbow segment in SH patients.

**Figure 3:**
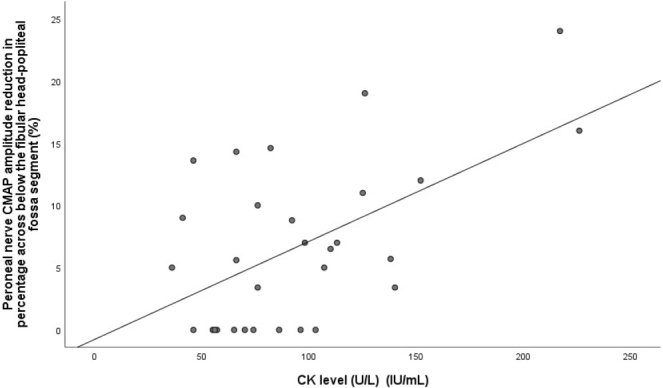
Correlation between CK level and peroneal nerve CMAP amplitude reduction in percentage across below the fibular head-popliteal fossa segment in SH patients.

Comparisons of sensory and motor nerve conduction study findings between the groups are presented in Tables 1 and 2, respectively. Nerve conduction studies were performed on the right/left upper and lower extremities in 22/8 SH patients and 24/16 healthy individuals (P = 0.245). Figure 1 illustrates the percentage reduction in peroneal nerve CMAP amplitude across the segment from below the fibular head to the popliteal fossa, comparing the two groups. Patients with electrodiagnostic findings suggestive of entrapment neuropathy are listed in Table 3. A negative correlation was observed between TPO antibody levels and both median nerve CNAP amplitude in the 2nd finger–wrist segment and ulnar motor NCV across the elbow segment (P = 0.004 r = −0.509/P = 0.022 r = −0.416) (Figure 2). A positive correlation was found between TPO antibody levels and F-wave latency in both the median and ulnar nerves (P = 0.028 r = 0.400/P = 0.005 r = 0.501). Additionally, a positive correlation was identified between T3 levels and ulnar motor NCV across the elbow segment, while a negative correlation was found between T3 levels and the NCV difference between the elbow and forearm segments (P = 0.032 r = 0.392, P = 0.031 r = −0.394). Figure 3 displays the positive correlation between CK levels and the percentage reduction in peroneal nerve CMAP amplitude across the segment from below the fibular head to the popliteal fossa segment (P = 0.021, r = 0.421).

**Table 1:** Comparison of sensory nerve conduction study findings between healthy individuals and patients with SH

Sensory nerve conduction study	Healthy individuals	SH patients	P value
Median nerve CNAP amplitude (μV)	41.8 ± 21.3 (39.6) (14.2-110.4)	44.0 ± 16.8 (43.1) (19.7-79.5)	0.336
Median sensory NCV- 2nd digit-wrist (m/s)	47.2 ± 4 (46.5) (41.1-58)	46.2 ± 4.5 (47) (36-53.2)	0.517
Ulnar nerve CNAP amplitude (μV)	44.7 ± 17.6 (42.9) (13.3-96.3)	51.2 ± 21.8 (51.1) (9.5-85.7)	0.149
Ulnar sensory NCV- 5th digit-wrist (m/s)	45.0 ± 4.3 (44.5) (39.1-56)	45.3 ± 4.4 (44.8) (36.7-55.7)	0.771
Sural nerve CNAP amplitude (μV)	19.2 ± 7.4 (18.5) (8.3-35)	17.0 ± 8.3 (15.4) (5.2-44.1)	0.194
Sural sensory NCV (m/s)	43.5 ± 5.6 (41.9) (35-56)	44.5 ± 6.2 (42.5) (35-63)	0.458
Superficial peroneal nerve CNAP amplitude (μV)	12.2 ± 4.3 (11.4) (5.3-20.9)	12.8 ± 5.3 (12.1) (6-28)	0.981
Superficial peroneal sensory NCV (m/s)	49.6 ± 6.1 (49.2) (39-64)	50.6 ± 7.3 (52.5) (35-60)	0.204

CNAP = compound nerve action potential; SH = subclinical hypothyroidism; NCV = nerve conduction study.

**Table 2:** Comparison of motor nerve conduction study findings between healthy individuals and patients with SH

Motor nerve conduction study	Healthy individuals Mean SD	SH patients Mean SD	P value
**Median nerve**			
*Distal latency (ms)*	2.9 ± 0.3 (2.9) (2.2-3.7)	2.9 ± 0.4 (2.9) (2.2-3.8)	0.664
*CMAP amplitude (mV)*	15.1 ± 5.1 (15.1) (5.1-26.7)	13.8 ± 4.7 (12.8) (7.6-24.2)	0.178
*Motor NCV- wrist-elbow (m/s)*	57.6 ± 4.9 (57) (50-67.3)	59.8 ± 4.6 (59.4) (52.1-71)	0.079
*F-wave latency (ms)*	25.9 ± 1.9 (26.1) (22.2-31.1)	26.1 ± 1.9 (25.8) (23-31)	0.905
**Ulnar nerve**			
*Distal latency (ms)*	2.2 ± 0.3 (2.2) (1.8-3)	2.2 ± 0.3 (2.2) (1.9-3.2)	0.633
*CMAP amplitude (mV)*	14.3 ± 3.1 (14.2) (8.8-21.6)	16.6 ± 7.7 (15.3) (11.2-56)	0.070
*Motor NCV- wrist-below elbow (m/s)*	63.2 ± 4.2 (63.4) (54.2-69)	64.5 ± 5.6 (65) (50-72.4)	0.121
*Motor NCV- below elbow-above elbow (m/s)*	59.1 ± 7.3 (59.5) (43-71)	60.0 ± 6.6 (60.7) (47-70)	0.605
*CMAP amplitude reduction in percentage- below elbow-above elbow (%)*	3.7 ± 4.3 (2.7) (0-14.9)	5.9 ± 5.8 (4.9) (0-17)	0.143
*Motor NCV difference between elbow segment and forearm segment*	4.1 ± 7.2 (5) (-11.9-16)	4.5 ± 6.1 (4.5) (-10.2-18.1)	0.934
*F-wave latency (ms)*	26.9 ± 3.7 (26.3) (23-45.8)	25.5 ± 2 (25.5) (22-29.6)	0.098
**Peroneal nerve-EDB**			
*Distal latency (ms)*	3.4 ± 0.5 (3.3) (2.7-4.5)	3.3 ± 0.5 (3.3) (2.3-4.5)	0.399
*CMAP amplitude (mV)*	9.4 ± 4.7 (8.8) (3.3-27.2)	8.2 ± 3.6 (7.7) (2.8-19.1)	0.262
*Motor NCV- ankle-below fibular head (m/s)*	50.3 ± 3.8 (48.5) (42.7-59)	52.1 ± 5.2 (51.4) (46.6-69.8)	0.137
*Motor NCV- below fibular head-popliteal fossa (m/s)*	52.2 ± 7.6 (50) (41.1-70)	52.8 ± 6.8 (53) (38.8-70)	0.304
*CMAP amplitude reduction in percentage- below fibular head-popliteal fossa (%)*	2.8 ± 3.5 (2) (0-15)	6.7 ± 6.6 (5.7) (0-24)	**0.017**
*Motor NCV difference between knee segment and leg segment*	-1.9 ± 8.6 (0) (-23.5-11)	-0.7 ± 4.7 (-1.7) (-8.5-13)	0.614
*F-wave latency (ms)* *	46.3 ± 2.9 (45.8) (42-52.5)	45.6 ± 2 (45.6) (42-49)	0.496
**Peroneal nerve-TA**			
*CMAP amplitude (mV)*	10.2 ± 3 (10.1) 4.0-17.2)	9.1 ± 3 (9.1) (3.7-14)	0.146
*Motor NCV- ankle-below fibular head (m/s)*	55.5 ± 9.5 (55) (41-72)	58.5 ± 8.2 (58.7) (44-71)	0.208
*CMAP amplitude reduction in percentage- below the fibular head-popliteal fossa (%)*	3.5 ± 4.2 (1.6) (0-13.9)	4.7 ± 7.1 (3) (0-34.9)	0.577
**Posterior tibial nerve**			
*Distal latency (ms)*	4.1 ± 0.8 (4.1) (2.7-5.9)	3.8 ± 0.8 (3.6) (2.3-6)	0.062
*CMAP amplitude (mV)*	14.1 ± 4.9 (13.3) (5.8-29)	12.4 ± 6.2 (10.8) (4.5-31)	0.047
*Motor NCV- ankle-popliteal fossa (m/s)*	46.2 ± 4.6 (45.6) (36-60)	48.0 ± 6.1 (48.3) (35.5-58)	0.154
*F-wave latency (ms)*	48.2 ± 4.1 (48.2) (42-59)	46.7 ± 3.5 (47.1) (40-56.1)	0.194

CMAP = compound muscle action potential; SH = subclinical hypothyroidism; NCV = nerve conduction study.

* F-wave was obtained in 34 healthy individuals and 24 patients with SH.

CMAP = compound muscle action potential; SH = subclinical hypothyroidism.

**Table 3:** Patients with electrodiagnostic findings suggestive of entrapment neuropathy according to neurophysiology tests

Patient	Age (years)/sex	Electrodiagnostic abnormality	Entrapment neuropathy according to neurophysiology tests
Patient 3	48/F	Median sensory NCV- 2nd digit-wrist (m/s)	CTS
Patient 5	42/F	CMAP amplitude reduction in percentage- below fibular head-popliteal fossa (%) – TA	PNFH
Patient 6	18/F	Peroneal Motor NCV difference between knee segment and leg segment -EDB	PNFH
Patient 11	36/M	Median sensory NCV- 2nd digit-wrist (m/s), Median nerve CMAP distal latency (ms)	CTS
Patient 13	64/M	Median sensory NCV- 2nd digit-wrist (m/s), Median nerve CMAP distal latency (ms), CMAP amplitude reduction in percentage- below fibular head-popliteal fossa (%) – EDB	CTS, PNFH
Patient 14	38/F	Peroneal Motor NCV difference between knee segment and leg segment -EDB	PNFH
Patient 27	43/F	Ulnar Motor NCV difference between elbow segment and forearm segment	UNE
Patient 30	49/F	Median sensory NCV- 2nd digit-wrist (m/s)	CTS

CMAP = compound muscle action potential; CNAP = compound nerve action potential; CTS = carpal tunnel syndrome; EDB = extensor digitorum brevis; NCV = nerve conduction velocity; PNFH = peroneal neuropathy at the fibular head; TA = tibialis anterior; UNE = ulnar neuropathy at the elbow.

CNAP = compound nerve action potential; NCV = nerve conduction velocity; SH = subclinical hypothyroidism; TPO thyroid peroxidase.

CMAP = compound muscle action potential; CK = creatine kinase; SH = subclinical hypothyroidism; TPO = thyroid peroxidase.

## DISCUSSION

In the present study, unlike other nerve conduction study findings, the reduction in peroneal nerve CMAP amplitude across the knee segment was greater in SH patients than in healthy individuals. Although the patients were asymptomatic, electrodiagnostic findings were consistent with CTS in four patients, PN in four patients, and UNE in one patient. Additionally, associations were observed between TPO antibody, T3, and CK levels and certain nerve conduction study findings.

The association between neuropathy—particularly CTS—and SH is well established.^4^ In the present study, subclinical CTS was also identified in some patients. However, the pathophysiology of CTS and other peripheral neuropathies in SH remains unclear. One proposed mechanism is mucinous infiltration of the tissues surrounding the nerve.^5,7,21^ Another possible explanation is impaired Na^⁺^/K^⁺^ pump function due to reduced ATPase activity in SH, which may disrupt axonal transport and lead to peripheral neuropathy.^5,7^ Although UNE is a common entrapment mononeuropathy, only one patient in this study showed electrophysiological findings consistent with subclinical UNE. This may suggest that UNE is a relatively rare manifestation in SH. Whether thyroid disease is a definitive risk factor for UNE remains unclear.^22^

The association between PNFH and factors such as weight loss, prolonged leg positioning (e.g., crossing the legs or squatting), and chronic diseases is well documented.^20,23,24^ In cases of PNFH related to weight loss, the peroneal nerve becomes more susceptible to entrapment due to the reduction of protective adipose tissue around the fibular head.^20,23,24^ Patients with SH may experience fluctuations in weight, which can similarly reduce the protective tissue surrounding the peroneal nerve.^20,23,24,25^ The observed positive correlation between serum CK levels and the reduction in peroneal nerve CMAP amplitude across the knee segment may suggest that this relationship is mediated by the loss of protective tissue in SH. However, the occurrence of PNFH in patients who have experienced weight loss but do not report prolonged leg positioning or trauma indicates that non-mechanical factors may also contribute to its development.^26,27^ It has been proposed that metabolic changes, such as disruptions in lipid metabolism associated with weight loss, may play a role in the pathophysiology of PNFH.^26,27,28^ Similarly, metabolic alterations in SH may lead to a deficiency of nutrients essential for nerve function, resulting in peripheral nerve damage.^7^ The peroneal nerve conduction abnormalities observed in this study could also be attributed to autoimmune mechanisms involving cytokines.^12^ Another possible explanation is mucinous infiltration of the tissues surrounding the nerve or impaired axonal transport caused by reduced Na^⁺^/K^⁺^ pump activity due to decreased thyroid hormone levels, as has been suggested in the pathogenesis of CTS in hypothyroid conditions.^5,7,21^

Reduction in peroneal nerve CMAP amplitude at the fibular head is a characteristic electrophysiological feature of PNFH. Most patients with PNFH who do not have chronic diseases and whose condition is associated with prolonged leg postures or weight loss typically show improvement.^20,23,24^ These features suggest that although partial axonal degeneration may occur in PNFH, demyelinating processes are more prominent. Both axonal degeneration and segmental demyelination have been implicated in the pathophysiology of subclinical hypothyroidism.^4,7,10,29,30^ The reduction in peroneal nerve CMAP amplitude across the knee segment observed in this study suggests that neuropathies associated with subclinical hypothyroidism may also exhibit prominent demyelinating features. Furthermore, the association of demyelinating neuropathies—such as acute inflammatory demyelinating polyradiculopathy—with subclinical hypothyroidism supports the findings of the present study.^29,30^

Although some studies have not identified electrodiagnostic abnormalities in patients with hypothyroidism, the majority suggest that both subclinical and clinical nerve involvement may occur.^4,7,10,31^ We believe that these discrepancies—or the observation that some studies report abnormalities in certain nerves while others do not—may be attributed to methodological differences. Factors such as disease duration, age, and sex could have influenced the results in both previous studies and the current one. Age and female sex have been reported as risk factors for hypothyroidism.^6^

In the present study, associations were found between nerve conduction study findings and TPO antibody, T3, and CK levels in SH patients. These results suggest that thyroid function tests, along with TPO antibody and CK levels, may be helpful in monitoring subclinical neuropathy. It has also been reported that as basal TSH levels increase, abnormalities in nerve conduction studies become more pronounced.^10^

This study has several limitations. The duration of SH varied between 1 and 30 months; however, it is important to note that the disease duration was less than 6 months in the majority of patients. BMI differed between healthy individuals and SH patients, which, while consistent with findings from previous studies may have influenced the results and thus represents a potential limitation. ^4,6,7^ Another limitation is that all SH patients were receiving treatment for hypothyroidism. We believe that comparative studies involving treated and untreated patients could offer valuable insights into the relationship between hypothyroidism and neuropathy, particularly regarding peroneal nerve conduction. Additionally, individuals with conditions known to predispose to polyneuropathy or entrapment mononeuropathy—such as diabetes mellitus—were not included as a separate group in this study. Including such individuals, both with and without subclinical hypothyroidism, in future research could help generate more comprehensive findings.

## CONCLUSION

This study demonstrated that subclinical CTS and PNFH may occur in SH, whereas UNE was not observed. It is recommended to avoid risk factors such as excessive use of the upper extremities—which may contribute to CTS—as well as weight loss and prolonged maintenance of the same leg posture, which may increase the risk of PNFH. Additionally, T3, TPO antibody, and CK levels may serve as useful markers for monitoring the subclinical effects of SH on peripheral nerves. However, further studies are needed to confirm the reliability and clinical significance of these findings.
